# Deep sequencing and miRNA profiles in alcohol-induced neuroinflammation and the TLR4 response in mice cerebral cortex

**DOI:** 10.1038/s41598-018-34277-y

**Published:** 2018-10-29

**Authors:** J. R. Ureña-Peralta, S. Alfonso-Loeches, C. M. Cuesta-Diaz, F. García-García, C. Guerri

**Affiliations:** 1 Molecular and Cellular Pathology of Alcohol Lab, Prince Felipe Research Center, Valencia, 46012 Spain; 2 Bioinformatics and Biostatistics Unit, Prince Felipe Research Center, Valencia, 46012 Spain

## Abstract

Alcohol abuse can induce brain injury and neurodegeneration, and recent evidence shows the participation of immune receptors toll-like in the neuroinflammation and brain damage. We evaluated the role of miRNAs as potential modulators of the neuroinflammation associated with alcohol abuse and the influence of the TLR4 response. Using mice cerebral cortex and next-generation sequencing (NGS), we identified miRNAs that were differentially expressed in the chronic alcohol-treated *versus* untreated WT or TLR4-KO mice. We observed a differentially expression of miR-183 Cluster (C) (miR-96/-182/-183), miR-200a and miR-200b, which were down-regulated, while mirR-125b was up-regulated in alcohol-treated WT *versus* (*vs*.) untreated mice. These miRNAs modulate targets genes related to the voltage-gated sodium channel, neuron hyperexcitability (Nav1.3, Trpv1, Smad3 and PP1-γ), as well as genes associated with innate immune TLR4 signaling response (Il1r1, Mapk14, Sirt1, Lrp6 and Bdnf). Functional enrichment of the miR-183C and miR-200a/b family target genes, revealed neuroinflammatory pathways networks involved in TLR4 signaling and alcohol abuse. The changes in the neuroinflammatory targets genes associated with alcohol abuse were mostly abolished in the TLR4-KO mice. Our results show the relationship between alcohol intake and miRNAs expression and open up new therapeutically targets to prevent deleterious effects of alcohol on the brain.

## Introduction

MicroRNAs (miRNAs) are a family of small non-coding RNA (about 21–25 nucleotides) that derives from endogenous long transcripts (pri-miRNAs), whose function as regulators of gene expression through translational repression has been demonstrated^1,2^. The central nervous system (CNS) is highly enriched with these small molecules and plays essential roles in neurogenesis, neural development, and synaptic plasticity^3^. However, the deregulation of miRNAs levels can lead to neurodegeneration and neurological disorders^4^. MicroRNAs are also considered important like-regulators of the immune system response^5^, but sustained aberrant expression levels have been associated with neuroinflammatory and immune-related neurodegenerative disorders, including multiple sclerosis^6,7^, Alzheimer’s disease (AD)^8^, Parkinson’s disease^9^ or neuronal hyperexcitability^10^.

In recent years, a new player has emerged in the field of alcohol abuse having demonstrated neuroinflammation participation in the neurotoxic and behavioral effects of alcohol. Studies in humans and experimental animals have revealed that alcohol alters the brain function and structure by contributing to alcohol dependence, and to behavioral, cognitive and psychiatric disorders^11^. Recent findings have shown the participation of the neuroimmune system response in not only alcohol-induced neurodegeneration, but also in alcohol consumption and addiction^12,13^. Indeed the activation of innate immune receptors, such as Toll-like (TLRs) and NOD-like receptors (inflammasome NLRs), appears to be an important target of alcohol actions on the brain neuroimmune response^11,14^. Experimental evidence indicates that alcohol can induce mitochondrial ROS generation and oxidative stress by promoting the TLRs and NLRs stimulation response and enhancing downstream pathways. The activation of transcriptional factors, such as nuclear factor *kappa* B (NF-κB) and interferon regulatory factor 3 (IRF3), triggers the induction of inflammatory genes and causes neuroinflammation along with behavioral dysfunctions^15^. Recent studies into amygdale synaptoneurosomes^16^ and the frontal cortex^17^ of mice with chronic alcohol intake have indicated the participation of miRNAs in the regulation of alcohol consumption and dependence. Some of these miRNAs have also been detected in the prefrontal cortex of post-mortem alcoholics^18^. Likewise, changes in miR-155 in the mice cerebellum^19,20^ have also been suggested to contribute to alcohol-induced neuroinflammation.

Our previous findings indicate the critical role of the TLR4 response in the neuroinflammatory response induced by alcohol and its contribution to brain damage and behavioral dysfunctions associated with alcohol abuse^14,21^. Therefore, by considering the influence of miRNAs as regulators of immune system, the aim of the present study is to explore the modulatory role of the miRNAs induced by chronic alcohol intake through the TLR4 response in the brain. To this end, we used cortices of WT and TLR4-KO with and without chronic alcohol treatment and the next-generation sequencing (high-throughput sequencing) technique to identify the miRNA profiles that could be differentially expressed. The bioinformatics pipelines identified several miRNAs and a regulatory pathways network related to the TLR4-response and immune system alterations. The results will provide a new direction for future applications of miRNAs alcohol diagnosis and treatment.

## Material and Methods

### Animals

Female wild-type (WT, TLR4^+/+^) (Harlan Ibérica S.L., Barcelona) and TLR4 *knockout* (TLR4-KO, TLR4^−/−^) mice, kindly provided by Dr. S. Akira (Osaka University, Japan) with C57BL/6 J genetic backgrounds, were used. Animals were kept under controlled light and dark conditions (12/12 h) at a temperature of 23 °C and at 60% humidity. Animal experiments were carried out in accordance with the guidelines set out in the European Communities Council Directive (86/609/ECC) and Spanish Royal Decree 1201/2005, and were approved by the Ethical Committee of Animal Experimentation of CIPF (Valencia, Spain).

### Alcohol treatment

For chronic alcohol treatment, 44 (11 animals/group) 7-week-old WT (C57BL/6 J) and TLR4-KO mice, weighing 18–20 g, were housed (4 animals/cage) and maintained with water (WT and TLR4-KO control) or with water containing 10% (v/v) alcohol. They were placed on a solid diet *ad libitum* for 5 months. During this period, daily food and liquid intake were similar for the WT and TLR4-KO mice, and for the alcohol-treated/untreated groups. Body weight gain at the end of the 5-month period was similar in both the WT (C57BL/6 J) and TLR4-KO mice treated with or without alcohol, as previously described^14^. The peak blood alcohol levels (BALs) obtained in mice 5 months after chronic ethanol treatment were around ≈125 mg/dl (range of 87–140 mg/dl) in the ethanol-treated WT mice, and about ≈ 122 mg/dl (range of 98–135 mg/dl) in the ethanol-treated-KO mice, respectively. The obtained BALs were similar to previously described ones^14^. After chronic alcohol treatment, mice were killed by cervical dislocation to remove and dissect the brain areas of interest by using the mouse brain atlas coordinates^22^, which were immediately snap-frozen in liquid nitrogen until used for further determination analyses.

### MicroRNA and total RNA isolation

Frozen cortex samples (100–200 mg) were used for the total and small RNA (sRNA) extraction; the manufacturer’s instructions were followed for the miRNeasy mini Kit (Appendix A, Qiagen, Hilden, Germany) with minor modifications. Using this protocol, the miRNA-enriched fraction that we obtained was enriched in various RNAs < 200 of nucleotides and depleted in rRNA. Briefly, 100–200 mg of tissue were disrupted with 1 ml of QIAzol (Qiagen, Maryland, USA), followed by the phenol chloroform method^23^. Total RNA and sRNA were isolated using the miRNeasy columns from the Qiagen Kit to obtain a separate sample for each RNA type. sRNAs were used for deep sequencing and the RT-qPCR miRNA evaluation. Finally, total RNA was used for the gene expression analysis.

### RNA Quantity and quality determinations

The quantities of each total RNA sample were determined using NanoDrop™, and quantity and qualities were measured in an Agilent 2100 bioanalyzer. Total RNA integrity was analyzed with the RNA Nano6000 kit (Agilent Technologies, Santa Clara, CA, USA) and the sRNA kit was employed for sRNAs (Agilent Technologies, Santa Clara, CA, USA). The best 9 samples for each condition were selected and combined in order to obtain 3-pooled samples for the 4 conditions generating a total of 12-pooled sRNA samples. After, we measured again the sRNA profiles with small-RNA kit (Agilent Technologies, Santa Clara, CA, USA) following manufacturer’s instructions and the total RNA integrity with the RNA Nano6000 kit (Agilent Technologies, Santa Clara, CA, USA).

### Small RNA library preparation

100 ng of the sRNA fraction from the pooled cortex samples were used to prepare the sRNA libraries with the Truseq library prep Small RNA Sample Preparation kit (Illumina, San Diego, USA). These samples were used for sequencing in HiSeq following the Illumina pooling manufacture’s guidelines. The cDNA from the miRNAs was obtained by the Superscript II Reverse Transcriptase kit (Thermo Fisher Scientific, Carlsbad, CA, USA) and unique indices were introduced during PCR amplification for 15 cycles. The sRNA libraries were visualized and quantified in an Agilent 2100 bioanalyzer. A multiplexed pool consisting of equimolar amounts of sRNA-derived libraries was prepared. Libraries were sequenced for 50 single reads cycles in HiSeq. 2000 (Illumina).

### Reverse transcription (RT)

2 µg of total RNA from cortical brain tissue were used. Samples were treated with *DNase I* (Invitrogen, Foster City, CA, USA) to avoid genomic DNA contamination. cDNA conversion was carried out with the High Capacity cDNA Reverse Transcription kit (Thermo Fisher Scientific, Foster City, CA, USA). The reaction was carried out in a Master cycler ep. 5341 (Eppendorf AG, Hamburg, Germany), performed at 25 °C for 10 min, then at 37 °C for 2 h and finally at 85 °C for 5 min.

### Reverse transcription (RT)-based TaqMan(®) MicroRNA assays

The expression levels of the selected miRNAs were confirmed by quantitative real-time PCR (RT-qPCR) using the TaqMan MicroRNA Assay kit for specific mature miRNAs and the TaqMan Advanced miRNA cDNA Synthesis kit (Applied Biosystems, Barcelona, Spain) following the manufacturer’s protocol.

### Real-time quantitative PCR

RT-qPCR was performed in a LightCycler*®* 480 System (Roche, Mannheim, Germany). The reactions contained *LightCycler 480 SYBR Green I Master (2×)* (Roche Applied Science, Mannheim, Germany), 5 μM of the forward and reverse primers and 1 μL of cDNA. The amplification efficiency (E) of primers was calculated from the plot of the Cq values against the cDNA input according to the equation E = [10(−1/slope)]. The relative expression ratio of a target/reference gene was calculated according to the Pfaffl equation^24^. Housekeeping cyclophilin-A (Ppia) was used as an internal control. The primer gene sequence is detailed in Table 1.

**Table 1:** The primer sequences of the genes used for real-time PCR.

Genes	Primer sequences (5′-3′)		Size (bp)
	Forward	Reverse	
SMAD3	*CAG GGC TTT GAG GCT GTC TA*	*GGT GCT GGT CACT GTCT GTC*	105
MAPK14	*GAC CGT TTC AGT CCA TCA TTC*	*AAC ACA TCC AAC AGA CCA ATC A*	100
PP1-Gamma	*GAG AAC GAG ATC CGA GGA CTC*	*CGT ATT CAA ACA GAC GGA GCA A*	142
TPRV1	*GCA GGA CAA GTG GGA CAG AT*	*TCG CCT CTG CAG GAA ATA CT*	235
BDNF	*ATT GGC TGG CGA TTC ATA AG*	*CTG TTT CCT TTC AGG TCA TGG*	250
Nav 1.3	*CAT TCA AAG GCT GGA TGG AT*	*TGA TGA CGC CGA TGA ATA GA*	159
SIRT1	*AGT TCC AGC CGT CTC TGT GT*	*CTC CAC GAA CAG CTT CAC AA*	198
LRP6	*CCA GGA ATG TCT CGA GGC AA*	*GCG ATG GTG GTG GGT TCA AA*	163
IL-1R1	*TGA AGA GCA CAG AGG GGA CT*	*CAT TGA TCC TGG GTC AGC TT*	169

### Real-time quantitative PCR miRNAs

RT-qPCR was performed in a LightCycler® 480 System (Roche, Mannheim, Germany). TaqMan and TaqMan Advance assays (Applied Biosystems) were used to quantify the specific miRNAs levels following the manufacturer’s protocol. The reaction contained 3.5 µl DEPC-treated Water (Thermo-Scientific), 1 µL of the cDNA template, 0.5 µL of the TaqMan miRNA Assay or the TaqMan Advance miRNA Assay primer, and 5 µL of the TaqMan Universal PCR Master Mix (Applied Biosystems). Sequences of the miRNA probes are specified in Table 2. miR-181a and miR-181b were used as internal controls.

**Table 2:** The sequences TaqMan miRNA assays used.

Probe	Region	Sequence
mmu-miR-183-5p	Chr.7: 129774905 - 129775014 [−]	*UAUGGCACUGGUAGAAUUCACU*
mmu-miR-351	Chr.X: 53053255 - 53053353 [−]	*UCCCUGAGGAGCCCUUUGAGCCUG*
mmu-miR-150*	Chr.7: 45121757 - 45121821 [+]	*CUGGUACAGGCCUGGGGGAUAG*
mmu-miR-1981	Chr.1: 184822407 - 184822488 [−]	*GUAAAGGCUGGGCUUAGACGUGGC*
hsa-miR-143	Chr.5: 149428918 - 149429023 [+]	*UGAGAUGAAGCACUGUAGCUC*
hsa-miR-9	Chr.1: 156420341 - 156420429 [−]	*UCUUUGGUUAUCUAGCUGUAUGA*
hsa-miR-21-5p	Chr.17: 59841266 - 59841337 [+]	*UAGCUUAUCAGACUGAUGUUGA*
mmu-miR-96	Chr.7: 129774692 - 129774769 [−]	*UUUGGCACUAGCACAUUUUUGCU*
mmu-miR-182-5p	Chr.6: 30165918 - 30165992 [−]	*UUUGGCAAUGGUAGAACUCACACCG*
mmu-miR-200a-5p	Chr.1: 1167863 - 1167952 [+]	*CAUCUUACCGGACAGUGCUGGA*
mmu-miR-200b-5p	Chr.1: 1167104 - 1167198 [+]	*CAUCUUACUGGGCAGCAUUGGA*
hsa-miR-125b	Chr.11: 122099757 - 122099844 [−]	*UCCCUGAGACCCUAACUUGUGA*
mmu-miR-7224-5p	Chr.2: 67675457 - 67675516 [+]	*GGGUAGGCCCCUCAGUGAAGA*
mmu-let-7b-5p	Chr.22: 46113686 - 46113768 [+]	*UGAGGUAGUAGGUUGUGUGGUU*

### Bioinformatics/Pipelines Analysis

#### Data pre-processing

A quality control analysis of the resulting fastq sequencing files was performed using FastQC, version 0.11.6^25^. Then the adapter sequences from Cutadapt version 1 were trimmed 8^26^. The trimmed reads were mapped against the databases for homologous non-coding RNAs (ncRNAs) with Bowtie, version 2.2.5^27^, and with version 2.1.0 of the TopHat software^28^. Normalization and post-alignment filters were used to remove low-quality reads, alignments characterized by sequencing errors or multiple mismatches. Read counts were generated using the Python and PERL programming software, which allows putative miRNAs of interest to be detected and annotated. After obtaining a counts matrix, the gene expression data were explored by the Principal Component Analysis and Clustering methods^29^. The raw counts data were normalized using Trimmed Mean of M values^30^.

#### Differential expression

The design was analyzed from the Bioconductor package edgeR^31^ by fitting a Negative Binomial Generalized Linear Model where the design matrix included one factor for all the experimental groups. This test detects the genes that are differentially expressed between experimental groups. The conventional multiple testing *p-value* correction procedure proposed by Benjamini-Hockberg was used to derive the adjusted p-values^32^.

#### Functional Profiling

The functional enrichment analysis allowed classes of genes or proteins to be identified that are over- or under-represented in a large set of genes or proteins, and could be associated with pathology. This step was carried out for the Gene Ontology (GO)^33^ terms using the Logistic Regression Model described by Sartor and colleagues^34^, which was adjusted^35–37^ to find out the functional blocks enriched under any of the conditions. Multiple testing was corrected by the Benjamini–Hochberg procedure^32^. We performed a complementary analysis for the selected microRNA clusters from DIANA-miRPath v3.0^38^ and the STRING database tools^39^.

### Statistical methods

We used SPSS version 17.0 and the R version 3.4.3 software^40^ for the validation analysis and bioinformatics. The Western blot and qPCR data were analyzed by the Student’s *t*-test. Differences at a value of P < 0.05 were considered statistically significant.

## Results

### Experimental design and the primary analysis of cortex miRNAs using NGS

**Figure 1:**
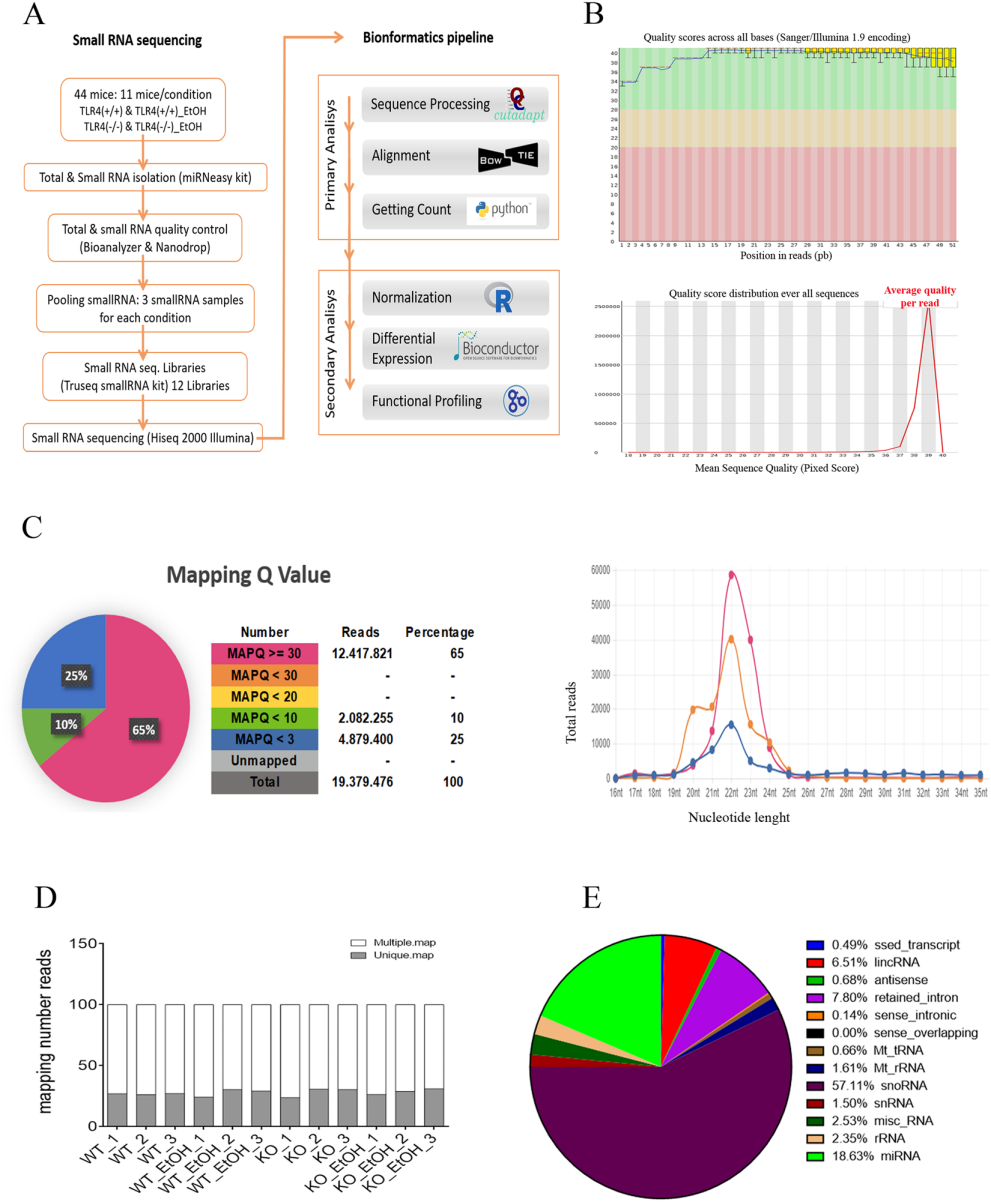
Experimental NGS Workflow and small RNA Quality Control. Small RNA libraries were prepared from the cortices of 44 mice (11 mice/condition). Samples were used for the deep sequencing protocol on the Illumina platform. Bioinformatics primary and secondary pipelines were used to detect the miRNAs profile and the differential expression miRNAs analysis **(A**). The Quality Control score, the Q value in all the nucleotides sequenced, were higher than 39, and the length with the best Q values was 51 nucleotides (**B**). Mapping Quality of the reads and distribution MAPQ by nucleotide length (21–25nt represent miRNA reads) (**C**). Number of reads aligned in multiple loci or unique loci in the reference genome sequence (**D**). Percentage of reads mapping RNA species (**E**).

The cerebral cortex is an important target brain area of alcohol’s actions^14^. To assess the miRNAs differentially expressed in cortices of the alcohol-treated and untreated mice, a deep sequencing analysis was used. Figure 1 shows the methodology scheme employed (Fig. 1A). For RNA isolation, 44 cortex samples were used, although only 36 of them had an optimal high quality (see Supplementary Table S1) for deep sequencing analysis. After checking the quality control of RNAs, 12 samples corresponding to the 3-pooled samples/condition (WT, WT + EtOH, TLR4-KO and TLR4-KO + EtOH) were generated. Three samples were pooled per condition to minimize the cost-efficient sampling strategy since some studies suggest that pooling minimizes the amount of information lost below the detection threshold^41^.

The bioinformatics’ analyses revealed the high quality control (QC) of the obtained sequences (Supplementary Table S2) after filtering data with the Cutadapt version 1.8^26^. For sequence mapping, short read alignments and the obtained sequences’ QC, Bowtie v.2.2.5^27^ and TopHat v.2.1.0^28^ were respectively used. Then, Python and PERL programming software was used, which allows putative miRNAs of interest to be detected following the GO (www.geneontology.org) (Fig. 1A).

The primary analysis of the distribution of quality read sequences^3^ indicated Q values above 39 in the 12 analyzed samples (Fig. 1B). In addition, the length distribution of sRNA reads obtained by NGS, revealed a consistent pattern with a large peak between 19–25nt, which corresponded to miRNAs (Fig. 1B). The diagram shown in Fig. 1C also illustrates the percentage of MAPQ quality against the non-coding sRNA database. This diagram indicates values up to 60% for MAPQ ≥ 30 in all the samples, which suggests an enrichment of the specific sRNAs. Finally, the percentage of the mapping distribution of the sequences showed that more than 70% of all the reads mapped back to multiple locations in the genome, which is an intrinsic characteristic of miRNAs^42^. The data in Fig. 1D also indicate that approximately 20% of sequences are distributed as single mappings in all the analyzed samples.

After deep sequencing, a total of [42.897.767 ± 417609] raw reads was identified from the 12 cortical samples. Once the trimmer was done, 95% of the sequences had adequate quality levels for further analyses, and a total of [41.461.124 ± 406609] trimming reads was obtained. The raw, trimmed, length and quality levels for the 12 samples also illustrated (Supplementary Table S1) that all the samples had Q-values above 39, which indicates a good Q score parameter for the sRNA reads. Data also indicate that around 19% of reads corresponded to miRNAs sequences from the total reads obtained in our NGS sequenced samples. Although the most abundant population was snoRNAs, the miRNAs reads that we obtained were enough to evaluate our study objective (Fig. 1E, Supplementary Table S3).

### The most abundant miRNAs in the cerebral cortex

Following our methodology scheme, we determined the miRNAs profile in cortex area by aligning reads to miRBase. Sequences were mapped against the database of the sRNAs by Burrows-Wheeler Alignment (BWA) to efficiently align the short sequences^43^. This yielded a total of 2.615.887 miRNA reads for the 12 samples (Supplementary Table S4).

**Figure 2:**
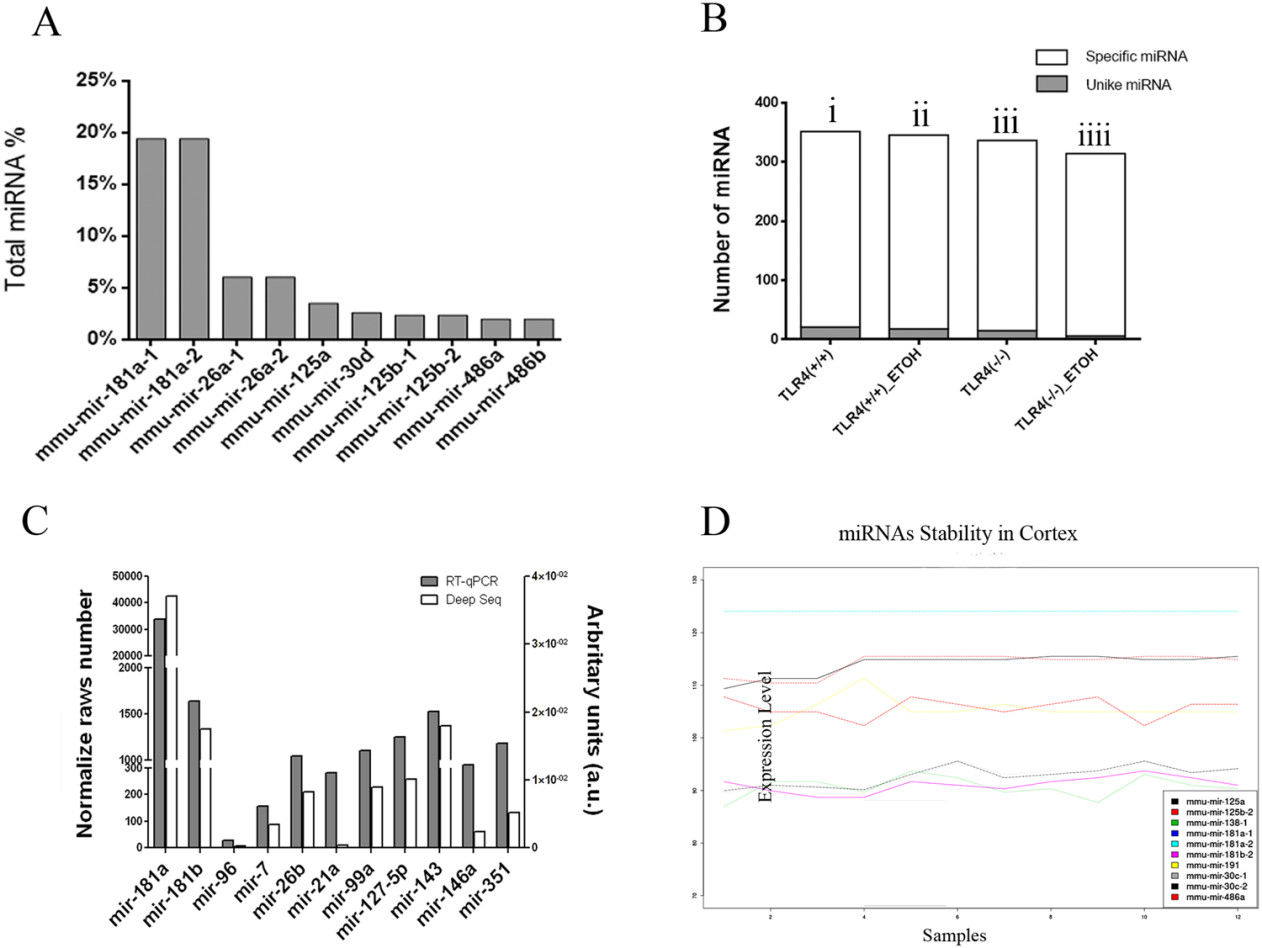
miRNA profile and differential expression analysis. The graph shows the most abundant miRNAs in mice cortex samples, where 50% of all the reads are identified as seven miRNAs (miR-181a, miR-26a, miR-30, miR-125a/b, miR-486a/b) (**A**). We showed the condition-specific patterns of the expression of the miRNAs in the mice cortex samples (**B**). Validation of high throughput data by sensitivity levels of the NGS and RT-qPCR techniques indicate that miR-181a is the most abundant miRNA species and miR-96 is the lowest in both protocols (**C**). Analysis of miRNAs’ stability under our specific conditions (**D**). The profile of the miRNAs with a differential expression in the three study comparisons (TLR4-KO + EtOH *vs*. KO; WE *vs*. WT; KO *vs*. WT) was assessed.

Figure 2A shows that the most abundant miRNAs expressed in the samples are: mmu-mir-181a, mmu-mir-26a, mmu-mir-125a, mmu-mir-30d, mmu-mir-125b, mmu-mir-486a and mmu-mir-486b. These miRNAs represent approximately 50% of the total miRNAs found.

We also identified 590 miRNAs, which were expressed in the cortex for all the used experimental conditions (Supplementary Table S5). Thus we found: i) 351 miRNAs expressed in the WT group, 21 of which were specific to this condition; ii) 345 miRNAs expressed in the WT + EtOH treatment, 18 of which were specific; iii) 336 miRNA in the TLR4-KO group, of which 15 were specific; iv) 314 miRNAs expressed in TLR4-KO + EtOH, six of which were specific for this experimental group (Fig. 2B).

Figure 2C shows the miRNAs levels validated by RT-qPCR TaqMan miRNA assays and we contrast NGS data (black bars) *vs*. TaqMan (white bars) values. The specific miR-181a was predominantly expressed in the brain cortex (Fig. 2A), while miR-96 had the lowest expression in NGS, as demonstrated by the TaqMan RT-qPCR (Fig. 2C). The results also indicated that, under our experimental conditions, RT-qPCR showed higher sensitivity than the NGS data. For instance, RT-qPCR signal for miR-96 was better than in NGS.

Using bioinformatics tools, we further assessed which miRNAs showed stable levels under the different experimental conditions to use them as internal controls for the RT-qPCR analysis. As shown in Fig. 2D, we chose mmu-mir-181a/-181b as housekeeper controls because these miRNAs were very stable in our samples.

### Differential miRNAs expression associated with alcohol treatment in the cortex

We also evaluated the miRNAs differential expression levels in our experimental groups. The number of reads/condition was distributed as follows: [701103 ± 20626] for WT; [633986 ± 101718] for WT + EtOH; [699894 ± 172107] for TLR4-KO and [580904 ± 79332] for TLR4-KO + EtOH. Table 5-S shows the differential expression of miRNAs in the three comparisons made: WT *vs*. WT + EtOH, WT *vs*. TLR4-KO and TLR4-KO *vs*. TLR4-KO-EtOH. The fold-change and p-value between comparisons are also included.

**Figure 3:**
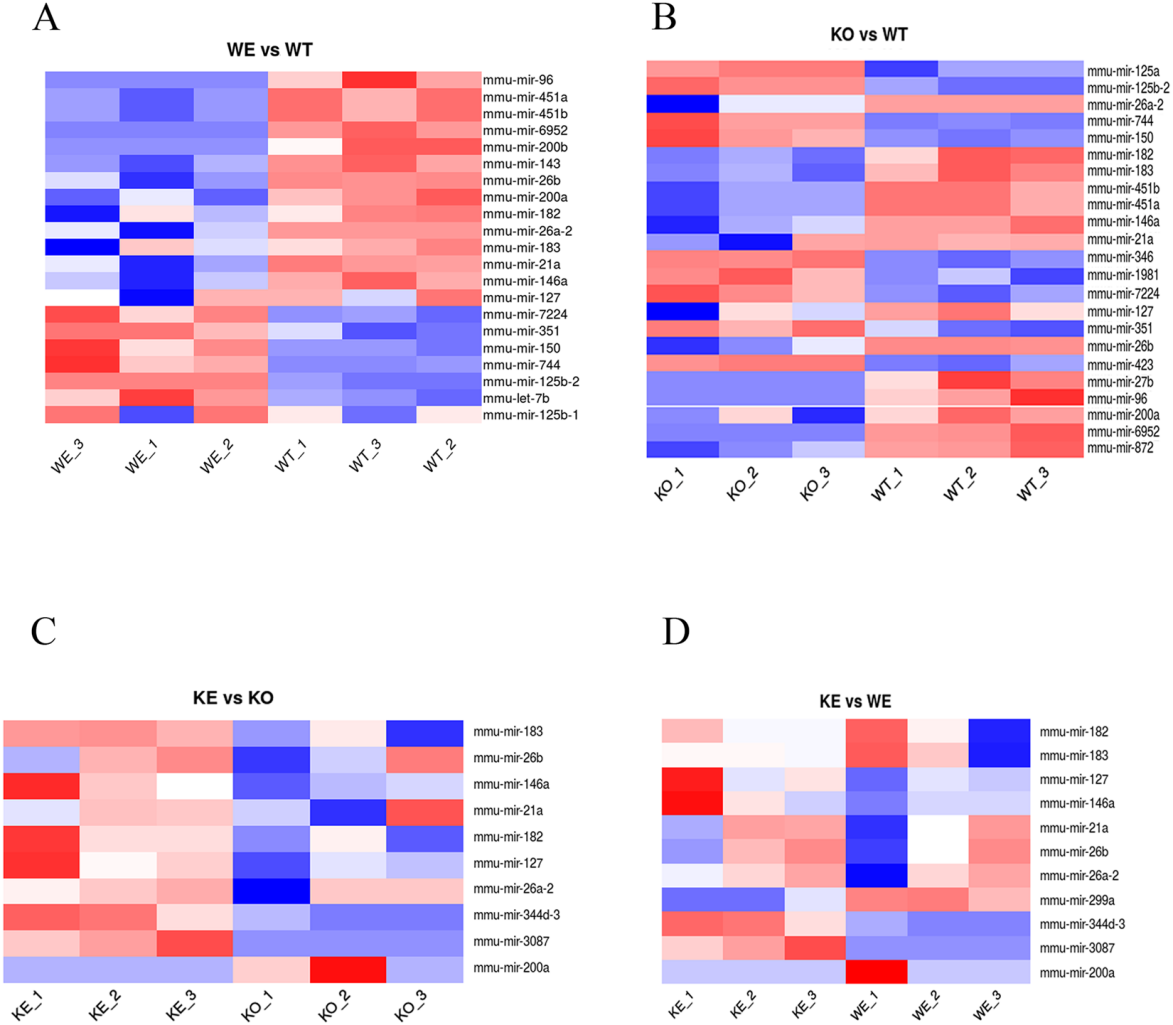
Heatmaps of cortical miRNA expression across alcohol-treated and untreated WT and TLR4-KO mice. The heatmaps show the miRNAs up-regulated (red) or down-regulated (blue) in each study comparison: WT + EtOH *vs*. WT (**A**), TLR4-KO *vs*. WT (**B**), TLR4-KO + EtOH *vs*. TLR4-KO (**C**) and TLR4-KO + EtOH *vs*. WT-EtOH (**D**).

Supplementary Table S6 and Fig. 3 show the differential profile of miRNAs in the four comparisons made. The data reveal that alcohol treatment up-regulated seven miRNAs, but lowered the levels of 14 miRNAs in the WT *vs*. the untreated control mice (Fig. 3A). We also noted that when the genotype comparison was made (TLR4-KO *vs*. WT), 14 miRNAs were down-regulated and nine were up-regulated, which indicates that the presence or absence of TLR4 is associated with changes in miRNA profiles in mice cortices (Fig. 3B); when the TLR4 receptor was absent, alcohol treatment only up-regulated eight miRNAs and down-regulated two miRNAs (Fig. 3C). Furthermore, the contribution of TLR4 under the ethanol condition (TLR4-KO-EtOH *vs*. WT-EtOH) shows an up-regulation of two miRNAs in TLR4-KO-EtHO and one down-regulated miRNAs (Fig. 3D).

**Figure 4:**
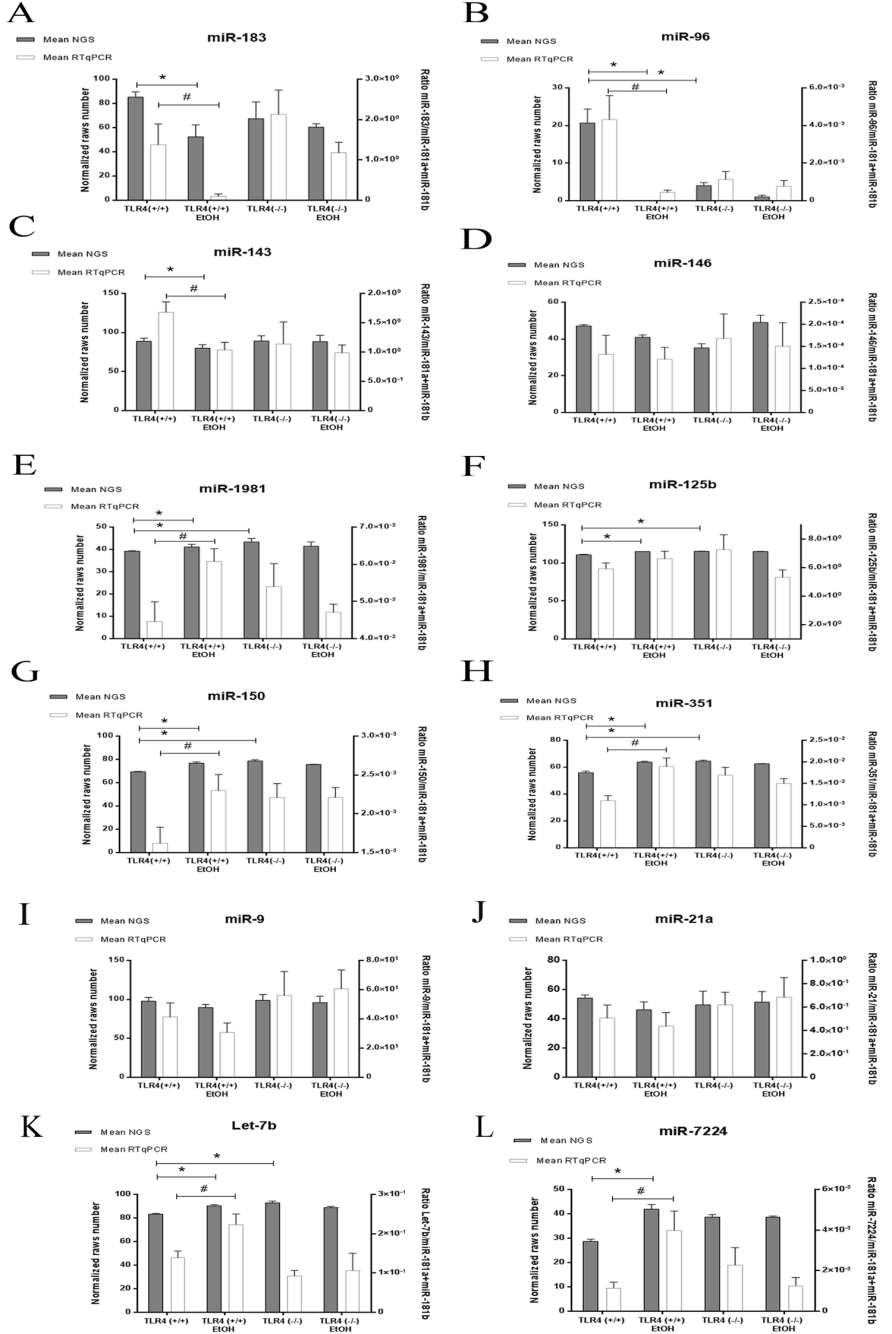
RT-qPCR and NGS differential expression analysis. The graphics represent the NGS and RT-qPCR comparisons made in the cortices of the WT and TLR4-KO mice, with or without ethanol treatment (**A**–**L**). The NGS black bars denote the mean of the miRNAs raw reads of three independent biological samples (right scale bar). White bars represent the mean ratio of miRNAs obtained by the RT-qPCR analysis in nine individual tissues. Data were normalized with miR-181a and miR-181b. Bars are represented as [mean ± SEM]. *p < 0.05, **p < 0.01, ***p < 0.001 for the NGS data, and ^#^p < 0.05, ^##^p < 0.01 (*Student’s t-test*).

In order to validate the NGS data obtained by bioinformatics platforms, we used TaqMan RT-qPCR to assess the relative expression of miRNAs. In particular, we assessed some miRNAs that were either dysregulated (up- or down-regulated) or were not changed by ethanol treatment. The bars in the Fig. 4 (A–L) illustrate how the alcohol treatment down-regulated some miRNAs (mmu-mir-183, mmu-mir-143 and mmu-mir-96), while others were up-regulated (mmu-mir-351, mmu-mir-150, mmu-mir-125b and mmu-mir-1981; mmu-mir-7224 and mmu-let7b) or simply did not change (mmu-mir-9 and mmu-mir-21a). It is noteworthy, that while miR-7224 present the highest fold-change value in the NGS analysis and the RT-qPCR data show an up-regulation tendency in the comparative WT *vs*. WE, the Ct values obtained with RT-qPCR show a very low amplification. The data also illustrate that although RT-qPCR and NGS have similar results, variations are observed (Fig. 4) in some miRNAs (e.g. mmu-mir-351 and let-7b).

The results in Fig. 4 also show that the alcohol treatment did not significantly affect most of the analyzed miRNAs when comparing TLR4-KO and TLR4-KO + EtOH. Nevertheless, a sharp drop in the expression of mmu-mir-96 in TLR4-KO was noted compared with the WT, which indicates that this miRNA could be a genotype-specific target (Fig. 4B). Likewise, a very significant reduction by the alcohol treatment in miR-96 expression was also noted (Fig. 4B). As mentioned above, both mmu-mir-181a and mmu-mir-181b were used as internal control.

### Alcohol deregulates clusters miR-183C and the miR-200s family

**Figure 5:**
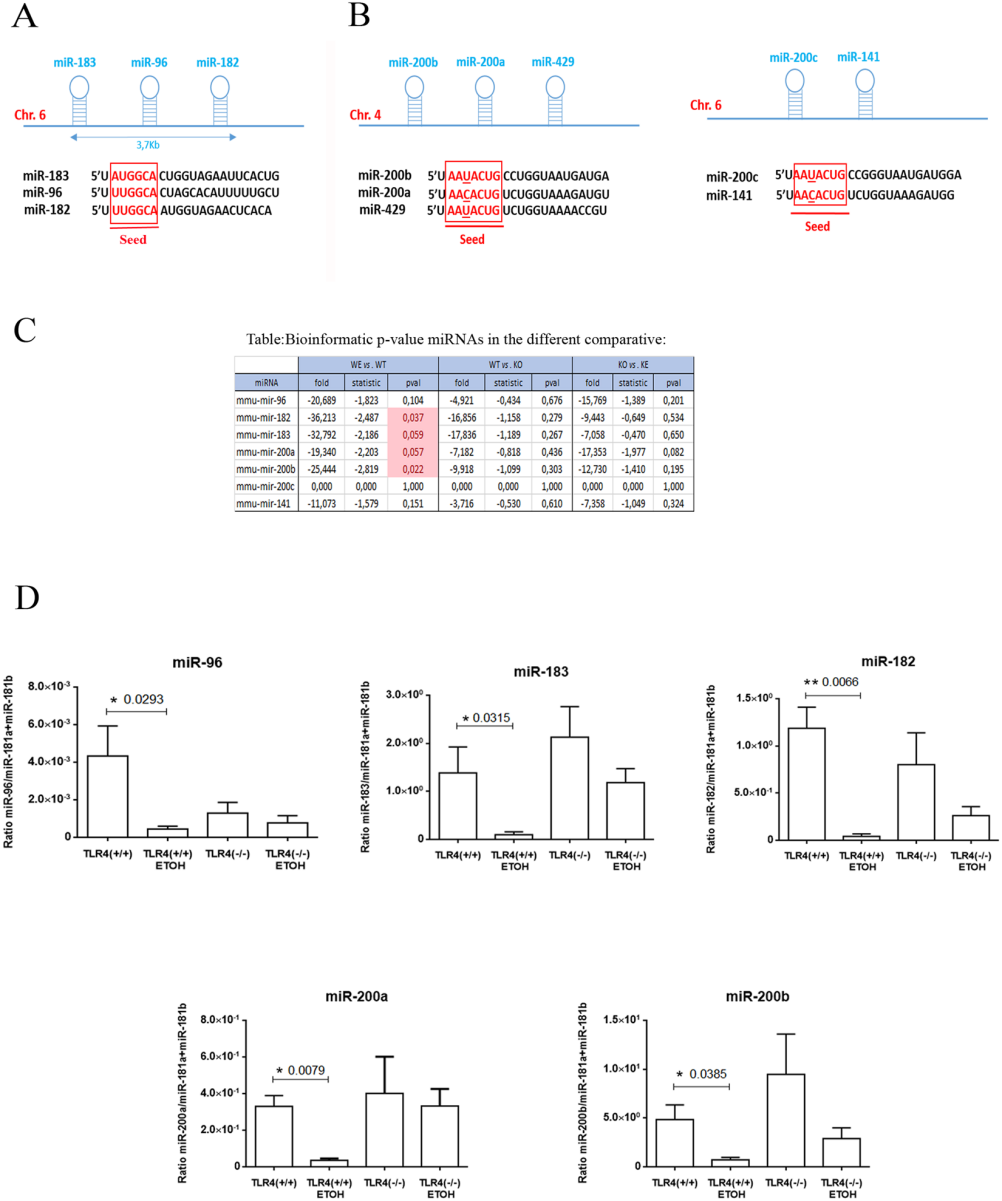
Genomic organization, structure and expression of miR-183 and the miR-200 gene clusters involved in alcohol abuse and the TLR4 immune response. (**A**) Homology sequence with a conserved seed^121^ of the miR components of the mmu-miR-183 cluster (miRs-183, -96, -182) on assembly mouse (Chromosome (Chr.) 6, Chr.7 human homolog). (**B**) The miR-200 family forms two clusters located in different genomic regions: cluster I miR-200s (miR-200b, -200a, -429) and cluster II miR-200s (−200c, −141) located in assembly mouse (Chr. 4 and Chr. 6, respectively) and divided into two functional groups based on their seed sequences^121^. (**C**) NGS data show that ethanol treatment causes a statistical significant expression (fold change, statistic, p-value (pval), p-value adjusted (padj)) of the selected miRs (miR-96, -182, -183, -200a and -200b). (**D**) The RT-qPCR shows a differential expression at the levels of miR-183, miR-182, miR-96 (miR-183C), miR-200a and miR-200b in the cortices of the ethanol-treated *vs*. untreated WT and TLR4-KO mice. n = 9–11 independent experiments. *p < 0.05, **p < 0.01 (*Student’s t-test*).

When we looked at the NGS and bioinformatics analysis results (Fig. 3), we noticed a group of miRNAs that play specific roles in neurological diseases, inflammation and immune disorders, which showed a systematic down-regulation in the cortices of the alcohol-treated WT mice. Moreover, the drop in the miRNAs in the TLR4-KO mice cortices was less marked, which indicated receptor influence. Figure 5A shows the structure, sequence and location of miR-96, miR-182 and miR-183. These miRNAs belong to a polycistronic miRNA cluster located in a 4-kilobase area in murine chromosome 6q (Fig. 5A)^44^.

The second miRNAs group to be studied was the miR-200s family. This group of miRNAs consists of five members organized into two clusters, miRs-200b/a/429 and miRs-200c/141 (Fig. 5B). The five miR-200s family components contain very similar seed sequences, as miR-200b/c/429 contains *AAUACU* and is located in chromosome 4, while the seed sequence of miR-200a/141 is *AACACU*, and is located in chromosome 6^45^. We therefore assessed and validated miR-183C (miR-96, -182 and -183), which is associated with neurological and autoimmune disorders^46^, and also miR-200a and miR-200b from the miR-200s family, which are involved in inflammatory pathways^47^. The data obtained by NGS and bioinformatics analysis revealed that alcohol treatment down-regulated these microRNAs (Fig. 5C). In particular, miRNA-96, miR-182, miR-183, miR-200a and miR-200b decreased -20.69-, -36.21-, -32.79-, -19.34- and -25.44 fold respectively, compared with the untreated WT controls (Fig. 5C). The validation of these miRNAs was done using new and independent RNA samples of the cortices from the WT and TLR4-KO mice, and with and without alcohol treatment, and the TaqMan miRNAs RT-qPCR analysis. The results obtained by the NGS data and the RT-qPCR validation confirmed that alcohol treatment down-regulated the miR-183C and the miR-200s family (Fig. 5D). We also confirmed that the absence of the TLR4 receptor attenuated the down-regulation effect induced by alcohol abuse.

### Interactome of miR-183C and the miR-200s family

**Figure 6:**
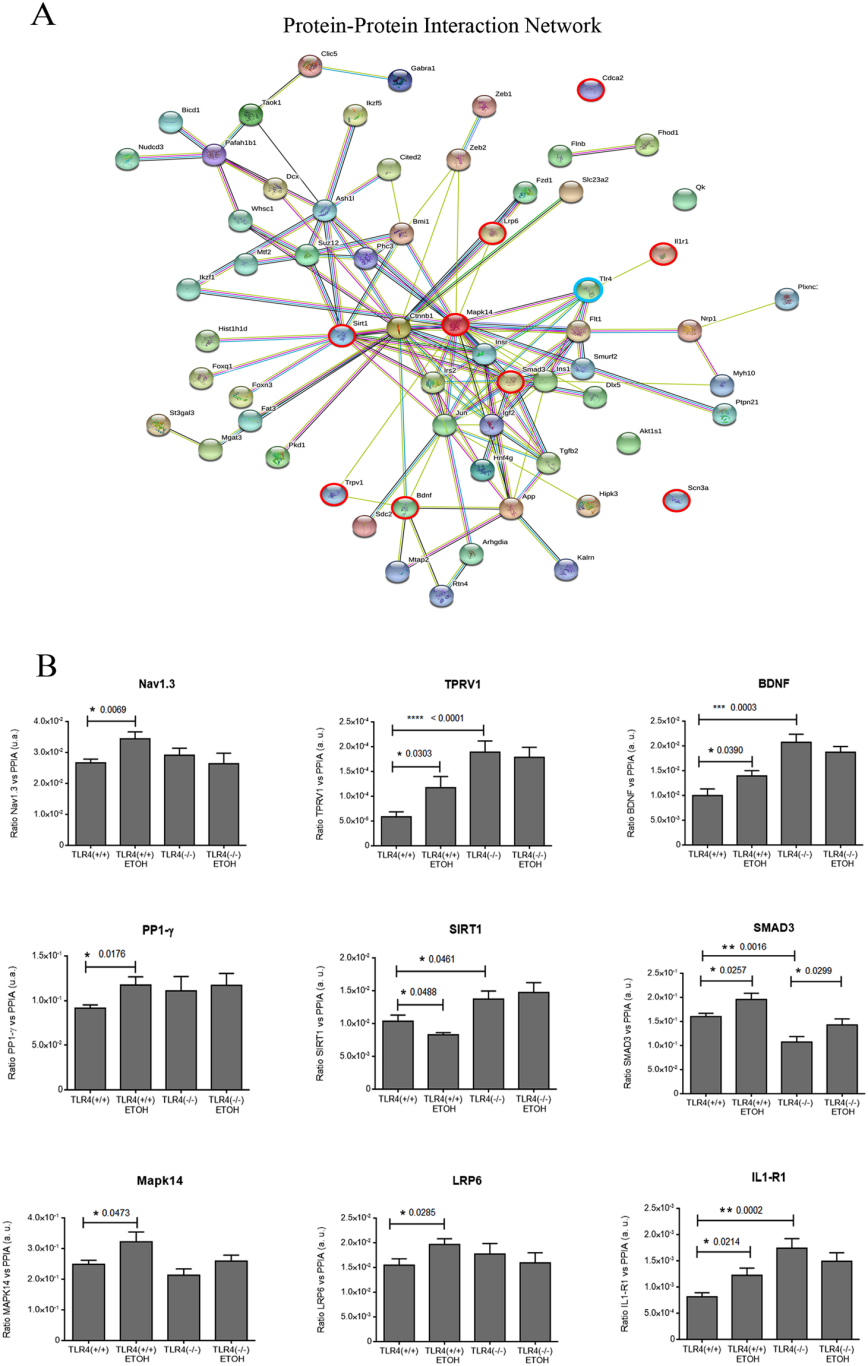
The interactome PPI (protein-protein interaction) network of miR-183C and the miR-200 family in alcohol-induced neuroinflammation in the WT and TLR4-KO mice cortex. (**A**) The diagram shows the different proteins regulated by miR183C and the miR-200 family, and the main proteins involved in alcohol effects (in red), including TLR4 (in blue). (**B**) The RT-qPCR results show the mRNA expression levels of Nav1.3, Trpv1, Bdnf, Cdca2, Sirt1, Smad3, Mapk14, Lrp6 and Il1r1. n = 10–11 independent experiments. *p < 0.05, **p < 0.01, ***p < 0.001, ****p < 0.0001 (*Student’s t-test*) (**B**).

We next assessed the potential genes/proteins targeted by miR-183C and miR-200a/b that were involved in the effects of alcohol abuse and TLR4 immune response. We firstly obtained target genes of these miRNAs from DIANA-miRPath v3.0 tool and then, we used the STRING database tool to predict the protein–protein interaction network. Figure 6A shows a significant interaction between proteins (p-value < 1.0e-16), which suggests that they are biologically connected as a group. This approach revealed a core group of 25 of the 63 more connected genes that are targets of these differentially expressed miRNAs, showing an evidence-based co-expression and/or co-localization.

Figure 6 revealed different genes regulated by miR-183C and the miR-200s family. Among these genes, we selected Nav1.3 (Scn3a), Bdnf, Tprv1, PP1-γ (Cdca2) and Lrp6, as they are involved in inflammatory response and neuronal pathologies^48–51^. Using RT-qPCR, we observed that alcohol treatment up-regulated the Nav1.3 expression mRNA levels in the WT mice cortices, but this up-regulation was not observed in the TLR4-KO mice (Fig. 6B). Alcohol treatment also up-regulated the mRNA expression levels of sodium channel Tprv1 in the WT mice, but did not affect the levels in TLR4-KO. However, the absence of the TLR4 receptor increased the Tprv1 gene expression levels in the cortex compared with the levels in the WT, indicating a genotype effect.

We next evaluated the expression of PP1-γ, Bdnf and Lrp6, which were also directly regulated by miR-183C. In particular, PP1-γ is a ubiquitous phosphatase in the CNS that participates in many brain functions by influencing the epigenetic state of these genes, particularly through post-translational modifications of histone proteins^52^ and the LRP6 gene, a low-density lipoprotein receptor-related protein 6 involved in endocytic processes^53^ and the modulation of inflammation via the WNT/β-catenin signaling pathway^49^. The results in Fig. 6B show that PP1-γ expression only increased in the cortices of the chronic alcohol-treated WT, but not of the TLR4-KO mice. In addition, chronic alcohol treatment significantly increased the levels of Bdnf and Lrp6 in the WT, but not in the TLR4-KO mice compared with their counterparts. However, we noted genotype differences in the expression levels of Bdnf.

We also checked Smad3, Sirt1, Mpak14 and Il1r1 as these genes are involved in neuroinflammatory signaling pathways and regulated by the miR-200a/b family. SIRT1 is involved in apoptosis processes, whereas SMAD3 has been associated with the TNF-α pathway^54^. Alcohol treatment increases the gene expression of Smad3 in the cortices of both the WT and TLR4-KO mice, while the same alcohol treatment reduced the gene expression of Sirt1 in the WT, with no effects on the TLR4-KO mice. We also observed that alcohol treatment up-regulated the gene expression of Mapk14 and the Il1r1 receptor in the cortices of the alcohol-treated WT mice, with no significant effects on TLR4-KO noted with the same alcohol treatment (Fig. 6B). It is noteworthy that the basal mRNA levels of Il1r1 were higher in the cortices of the TLR4-KO than in the WT mice, which suggests some genotype effect that might compensate the absence of TLR4.

### The functional enrichment of the miR-183C and miR-200a/b family target genes identified the molecular pathway networks in alcohol abuse

**Figure 7:**
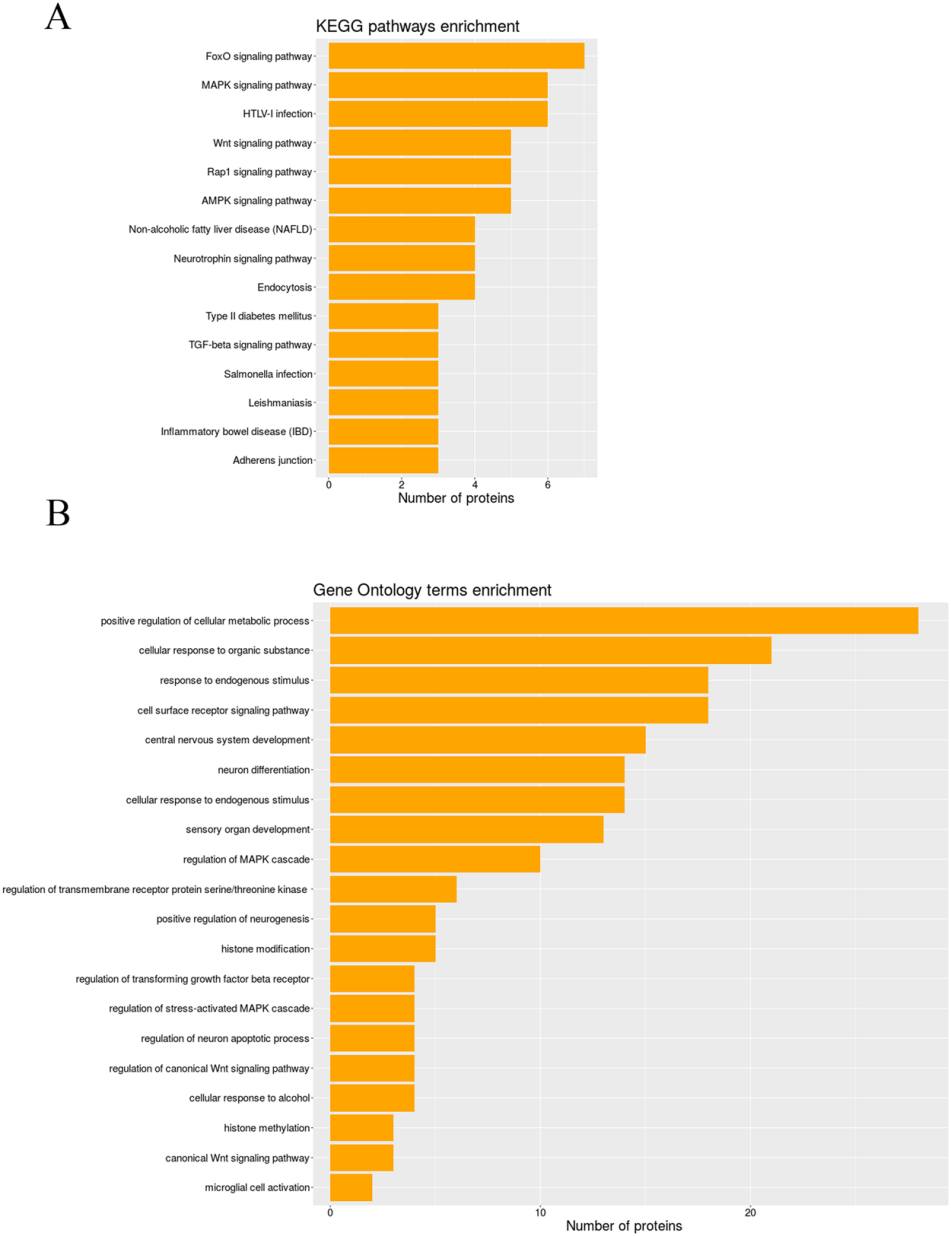
KEGG pathway and GO enrichment analysis of the differential expressed miR-183C and the miR-200s family in alcohol abuse. (**A**) The top 15 most enriched KEGG pathways are shown. All the coding functions are annotated against the KEGG database of miR-183C and miR-200s family, which show regulatory pathways, including the functional networks of these clusters. (**B**) The top 20 enriched GO framework were calculated and plotted and showed the molecular gene functions associated with biological processes and their relationships with miR-183C and the miR-200 family.

We next performed a complementary analysis to further establish the main biological functions and key pathways involved in alcohol abuse, TLR4 immune response and miR-183C/miR-200s family (Fig. 7). For this objective, the STRING bioinformatic tool was used to perform the functional enrichment. The Gene Ontology (GO) and Kyoto Encyclopedia of Genes and Genomes (KEGG) pathway enrichment analysis are major processes used to investigate the gene groups that participate in common biological responses or acquire related functions. The KEGG pathway enrichment analyses show that the top 15 pathways were enhanced, including the MAPK-, AMPK-, FoxO-, TGF-beta- and Wnt- signaling pathways, endocytosis processes, non-alcoholic fatty liver disease and viral infection, among others (Fig. 7A). Moreover, the enriched GO terms selected the top 20 enriched biological processes, such as cellular response to alcohol, bacterial invasion, apoptosis, MAPK cascade regulation, canonical Wnt signaling pathway, microglial cell activation and histone modification, etc. (Fig. 7B).

The above functional pathways (Fig. 7) are highly involved in the regulatory role of miR-183C and the miR-200s family in chronic alcohol abuse. Indeed there is evidence to indicate that miR-200b/c exerts a protective effect by targeting ZEB1/2, which may be associated with the inhibition of the p38 MAPK and TGF-β/Smad3 signaling pathways^55^. We also observed that other important pathways, such as the FoxO and MAPK signaling pathways, were retrieved, play important roles in apoptosis and neuron development functions, and are regulated by some evaluated genes, such as Mapk14, Sirt1, Smad3 and Bdnf. Additionally, TRPV1 stimulation has been shown to lead to the activation of microglia and astrocytes^56^, and the induction of TRPV1 in sensory neurons is also associated with mechanical hypersensitivity after nerve injury via c-Jun N-terminal kinase (JNK) and p38 MAPK^57^.

Furthermore, pathway interactions are observed in Table 6-S, and specifically some of these pathways might interact through a gene or a combination of genes. For instance, the MAPK- and FOXO- signaling pathways might interact via phospho-P38 (MAPK14). The detailed information of the genes enrichment in the KEGG pathways and GO terms is provided in supplementary Table S7.

In short, the results highlight that the regulatory gene-processes controlled by both miR-183C and the miR-200s family are directly involved in the TLR4 innate immune signaling response associated with neuroinflammation and brain damage.

## Discussion

We and others have demonstrated the critical role of the innate immune system and the TLR4 signaling response in ethanol-induced neuroinflammation^14,20^ and neurodegeneration associated with neural death^14^, myelin disruption^58^ and impairment in the proteolytic pathways (ubiquitin-proteasome and autophagy-lysosome)^59^. As miRNAs are important regulators of gene expression, and play a key role in neurodegenerative diseases^60^, immune response and neuroinflammation^61^, our goal was to evaluate the potential modulatory role of miRNAs in the alcohol-induced immune TLR4 response and neuroinflammation in the cerebral cortex. By conducting NGS along with bioinformatics to identify the miRNAs affected by chronic alcohol consumption and the role of TLR4 response in the mouse cerebral cortex, we identified some miRNAs that were differently expressed in the cortices of the chronic alcohol-treated mice, targeted neuronal excitability, TLR4 signaling response and inflammation. In particular, we identified a set of miRNAs clusters, miR-183C (miR-183, -96, -182) and the miR-200s family, which regulate target genes such as MAPK and IL1-R1, and were up-regulated in the cerebral cortices of the WT mice, but not in TLR4-KO. These findings indicate the specific role of TLR4 signaling in alcohol-induced miRNAs expression.

MiRNAs are abundantly expressed in the brain^60,62,63^ and play a major regulatory role in gene expression due to the ability of a single miRNA to bind multiple mRNAs^64^ by allowing the cell to quickly adapt to new environments. Several studies have demonstrated that alcohol abuse deregulated miRNAs, and changes in miRNAs have been identified in post-mortem frontal cortices and other regions of human alcoholics^18,65^, and even in the cortices of alcohol-dependent mice^17,66–68^. For instance, miR-152, let-7, mirR-15, miR-140 and miR-7, among others, have been detected in the prefrontal cortices of human alcoholics and mice with chronic alcohol consumption^18^. Our study also identified several miRNAs (miR-183C, miR-96, miR-451, miR-200, miR-182, miR-146, miR-127 and let-7b) that were up-regulated in mice cerebral cortices in early stages of alcohol dependence^17^, while we observed the down-regulation of these miRNAs after chronic alcohol consumption, except the let-7b that was up-regulated. Adaptations of miRNAs after long-term alcohol intake (5 months *vs*. a short term of 20 days), along with differences in both the alcohol paradigm and the methodology used to assess miRNAs (NGS *vs*. microarrays)^69^, might explain the observed differences. Other miRNAs, such as miR-411, have been associated with alcohol consumption^70^, while microglia-derived let-7b has been suggested to contribute to alcohol-induced neuroimmune pathology^71^. A recent study using microarrays from amygdala synaptoneurosomes (SN) of chronic alcohol drinking mice and a bioinformatics analysis^16^ has identified some microRNA–mRNA synaptic interactions that could participate in the aberrations in synaptic plasticity in alcoholics. This study also identified the alcohol-responsive miRNAs that were correlated with astrocytic, microglial and neuronal modules^16^. Unfortunately by using our experimental approach and the all cerebral cortices, we were unable to detect the ethanol-sensitive miRNAs, associated with synaptoneurosomes, glial cells or neurons, as previously reported. Nevertheless, our main aim was to identify the cortical miRNAs associated with neuroinflammatory actions of alcohol linked to the TLR4 signaling response.

Using high-throughput data, along with an integrated bioinformatics analysis of the mice cortex, we identified that the 50% of the raw miRNA in our samples corresponded to seven miRNAs (mmu-miR-181a, mmu-miR-26a, mmu-miR-125a, mmu-miR-30d, mmu-miR-125b, mmu-miR-486a and mmu-miR-486b). Some of these miRNAs, such as miR-181a, have been linked to the pathogenesis of multiple sclerosis and TNF-α signaling^72,73^, or miR-125 that controls functional synaptic integration and is involved in the modulation of innate immune signaling^74,75^. However, when the differential expression of the miRNAs associated with chronic alcohol treatment was evaluated, we noted that while 14 miRNAs were down-regulated, seven were up-regulated compared to the alcohol-treated and untreated WT mice. In the other comparisons, we observed that nine miRNAs were up-regulated, but only one miRNAs were down-regulated between the TLR4-KO + EtOH and TLR4-KO mice, with alcohol having less influence on mice with no receptor (see Table 5-S).

Consequently, although we obtained several miRNAs that were deregulated by chronic alcohol abuse, when we looked at the raw and fold changes in the differentially expressed miRNAs by alcohol treatment, we identified and focused our study on the cluster miR-183C (miR-182/96/183), which was down-regulated in the cerebral cortices of the WT mice, but no significant differences were noted in the TLR4-KO mice, although a genotype effect was observed in some miRNAs (e.g. mmu-miR-96). Interestingly, the cluster miR-183C represents a gene family with a homology sequence^46^ that is involved in several pathologies, such as autoimmune diseases^76^, neurological and neurodegenerative disorders^77,78^, neuropathic pain^79^ and retinal degeneration^80^. Notably, the miRNAs that make up this aforementioned cluster, miR-183C, were also deregulated in the mouse frontal cortex in early alcohol addiction stages^17^.

Our results further demonstrated that chronic alcohol intake down-regulated the miR-200s family (miR-200a/b) in the cerebral cortices of the WT mice, while the same alcohol treatment only significantly affected the expression levels of these miRNAs in the cortices of the TLR4-KO mice. The miR-200s family is involved in the TLR4/NF-κB inflammatory signaling pathway^81^, modulates microglia-induced neuroinflammation *via* the cJun/MAPK signaling pathway^82^, and also plays a role in neuropathic pain^83^. Therefore, some authors have described the induction of the genes related with neuroimmune disorders^84,85^, microglial activation and neuroinflammation^14,86^, TLR4/NF-κB inflammatory signaling^11,14^ and neurodegeneration^85^, as observed in humans and animal models with chronic alcohol abuse. Therefore, the down-regulation of miR-183C and the miR-200a/b family is expected to trigger the above processes. Other studies have further demonstrated that the induction of miR-155 in the cerebellum of mice with chronic alcohol intake contributes to neuroinflammation. Notably, these effects were dependent on the TLR4 response because alcohol-fed TLR4-KO mice were protected from the induction of microglia miR-155, which supports the role of the TLR4 signaling response in alcohol-induced neuroinflammation^20^.

Evidence indicates that the miR-200s family and miR-183C can work together. Indeed the STRING database tool predicted a common protein–protein interaction network (Fig. 6A). These miRNAs family members coordinate neural induction from human embryonic stem cells^87^ by contributing to the end-point of prion disease^88^, or to brain damage after virus infection^47^. MiR-200 and miR-182 also participate in ischemia by up-regulating their expression after ischemic preconditioning and being neuroprotective in later stages^89^.

MiRNAs have the ability to affect different targets by base pair complementarity^90^. When we looked at the targeted-genes regulated by both clusters, miR-183C and the miR-200s family, and by using a functional-enrichment that allowed us to identify classes of genes that are over- or infra-represented in associated pathologies (see Fig. 6), we selected some target genes that were regulated by miR-183C, such as Nav1.3, BDNF and TPRV1. Consequently, we found that chronic alcohol abuse increased the expression of these genes in WT mice cortices, with minor changes in TLR4-KO compared with the untreated control mice.

Early studies also have demonstrated that a high anesthetic alcohol concentration inhibits sodium channel Nav1.3^91^, although lower alcohol levels, which occurs in chronic alcohol treatment, can promote adaptation by increasing its expression levels since the up-regulation of Nav1.3 is associated with traumatic brain injury severity in rats^92^. The other gene is vanilloid receptor TRPV1, a multimodal ion channel of afferent neurons that seems to play a role in hyperalgesia associated with inflammation^93,94^. Alcohol potentiates the response of TRPV1, while the studies of Hirota *et al*. suggest alcohol-induced sensory responses of inflamed tissues^95^. This channel is also involved in specific behavioral actions of alcohol as the deletion of TRPV1 in mice alters behavioral effects of alcohol^96^.

Another target gene regulated by miR-183C is BDNF, an important modulator of synaptic plasticity that is associated with signaling mechanisms and acts in specific brain circuitry by regulating synaptic plasticity related to alcohol drinking behaviors^97^. Indeed miR-206 in medial frontal rat cortices has been suggested to regulate BDNF and alcohol drinking^98^, while BDNF polymorphism increases compulsive alcohol drinking in mice^99^. It was noteworthy that the levels of BDNF mRNA were not affected by ethanol treatment in TLR4-KO mice since these mice had higher BDNF levels than the WT mice, which indicates some genotype differences. As BDNF is a neuroprotective agent^100,101^, an increase in this trophic factor might confer some protection against ethanol-induced neuropathology, as reported in previous studies^14,58^, but does not affect alcohol drinking behavior^102^.

Likewise, LRP6 is a Wnt-protein binding regulated by miR-183 that contributes to regulate gluconeogenesis, insulin secretion and diabetes^103^. LRP6 is a component of the Wnt-Fzd-LRP5-LRP6 complex that triggers the Wnt/β-catenin signaling pathway, which can modulate the TNF-α-induced inflammatory response. Indeed Wnt- and NF-kB-signaling are inter-connected because recombinant Wnt proteins induce NF-kB nuclear translocations and their target DNA binding^49^. Some studies indicate that prenatal alcohol exposure suppresses Wnt signaling in differentiating human NSC by contributing to fetal alcohol spectrum disorders^104^, although we found that LRP6 increased in the cortices of the chronic alcohol treated-WT mice (Fig. 6B). Other functions denote LRP6 as a cell surface endocytosis receptor^53^, which has also been associated with Alzheimer’s disease^105^.

Finally, protein phosphatase PP1-γ is a ubiquitous phosphatase in the brain that participates in many functions, including regulation of the genes involved in memory formation by influencing the epigenetic state of these genes through post-translational modifications of histone proteins^52^. Recent findings have shown that PP1-γ involves miR-183C and its selective regulation during memory formation^106^. Strikingly, a recent study into human alcohol binge-drinking indicates that inhibition of PP1-γ correlates with the suppression of TLR4/TRIF activation in macrophages^107^. An association has also been found between hypermethylation in other nuclear gene phosphatases, such as the PPM1G gene locus, and early escalation of alcohol use and impulsiveness in individuals with alcohol-use disorders^108^.

The present findings provide further evidence that the genes regulated by the miR-200a/b family, such as SIRT1, MAPK14, SMAD3 and IL1-R1, are up-regulated in the cortices of WT mice with chronic alcohol consumption, and have little or no significant effect on their expression in alcohol-treated TLR4-KO mice cortices, although IL1-R1 expression shows some genotype effects, as revealed by this receptor’s increased gene expression, which might occur to compensate the absence of TLR4. These target genes are suggestive of being important in the modulation of alcohol actions on the brain, and are involved in neuroimmune signaling and circadian rhythm. For instance, p38 MAPK (MAPK14) is involved in TLR4/IL-1R1 pathways, and leads to pro-inflammatory cytokines production^109^. Several studies indicate that the p38 MAPK signaling pathway regulates alcohol-induced neurodegeneration^110^. Similarly, alcohol activates IL1-R1^111,112^ by triggering the same signaling pathways as the TLR4 response. Indeed, TLR4 leads to the production of cytokines and inflammatory mediators, which participate in neuroinflammation, brain damage and behavioral response associated with alcohol consumption^11,14,113,114^. The administration of anti-inflammatory compounds or blocking TLR4 greatly ameliorates the neuroinflammatory effects of alcohol^115,116^. Chronic alcohol intake also increases IL-1β in the brain^14,20^, and genetic polymorphisms in IL-1β and IL-1RN (antagonist) are associated with the risk of alcohol dependence in humans^117,118^.

Another gene regulated by the miR-200s family is SIRT1, which encodes a family of highly conserved NAD^+^-dependent deacetylases that act as cellular sensors to detect energy availability and to modulate metabolic processes. SIRT1, the best-studied member of the mammalian sirtuins, is expressed in the brain and is a crucial component of the multiple interconnected regulatory networks that modulate dendritic and axonal growth. SIRT1 modulates synaptic plasticity through the regulation of BDNF^119^, being also a neuroprotector against aging and controlling the energy metabolism and circadian rhythm^120,121^. Accordingly, some studies demonstrate that alcohol interacts with circadian rhythm by affecting the biological clock, while some genes, like PER2, promote alcohol consumption^122^. Alteration to several circadian clock genes has been reported in chronic alcoholics^123^. Strikingly, we observed how TLR4-KO showed some genotype effects since the SIRT1 levels were higher than in the WT mice. Higher levels of both BDNF and SIRT1 in TLR4-KO mice could promote protection against ethanol-induced neuropathological changes.

In summary, the present results highlight new putative miRNAs in the cerebral cortex that are involved in alcohol-induced neuroinflammation and the TLR4 signaling response. The NGS data reveal that miR-183C and the miR-200s family could modulate the neuroinflammatory pathways associated with alcohol abuse. We further present enriched GO and KEGG functional networks analyses that open up possible new therapeutically gene targets to prevent the deleterious effects of alcohol abuse linked to the TLR4 immune response in the brain.

## Electronic supplementary material

Legends Supplementary Tables

Supplementary Tables 1S-7S

## Data Availability

The data discussed in this publication have been deposited in NCBI’s Gene Expression Omnibus^124^ and are accessible through GEO Series accession number GSE120373 (https://www.ncbi.nlm.nih.gov/geo/query/acc.cgi?acc=).
