# Supplementary material for: Deep sequencing and miRNA profiles in alcohol-induced neuroinflammation and the TLR4 response in mice cerebral cortex
Source: Sci Rep. 2018 Oct 29;8:15913. doi: 10.1038/s41598-018-34277-y (PMC6206094; doi:10.1038/s41598-018-34277-y)
Supplement: Supplementary file 1 — Legends Supplementary Tables [file 41598_2018_34277_MOESM1_ESM.pdf]

Deep sequencing and miRNA profiles in alcohol-induced neuroinflammation and the TLR4 response in mice cerebral cortex

Ureña-Peralta JR[1], Alfonso-Loeches S[1], Cuesta-Diaz CM[1], García-García F[2], Guerri C[1, #].

[1]Molecular and Cellular Pathology of Alcohol Lab, Prince Felipe Research Center, Valencia 46012, Spain

[2]Bioinformatics and Biostatistics Unit, Prince Felipe Research Center, Valencia 46012, Spain.

***# Correspondence to*:**

Consuelo Guerri

Molecular and Cellular Pathology of Alcohol

Prince Felipe Research Center

C/ Eduardo Primo Yúfera 3, Valencia 46012, Spain

Tel: +34 96 3289680, Fax: +34 96 328 9701.

E-mail: guerri@cipf.es

Supplementary Tables Legends

Supplementary Table 1S: Total RNA Quality Control: Total RNA were isolated from cortex mice and measured with nanodrop for obtain the protein contamination with 260/280 ratio and the compounds contamination with 260/230 ratio. The total RNA amount and RIN values were obtained with bioanalyzer Agilent 2100. The nine better samples in each condition were selected for NGS study.

Supplementary Table 2S: Deep Sequencing reads Quality Control: After Illumina HiSeq sequencing protocol was done, the raw reads were evaluated. The table show the number of sequences obtained in each pool sample, the mean length of the reads, the average of total Mega Bases information obtained and the mean of sequencing quality. These results were defined before and after trimming process.

Supplementary Table 3S: Non-coding small RNA Percentage summary: Trimmed reads were mapped against databases for homologous non-coding RNAs (ncRNAs) with Bowtie version 2.2.5 and TopHat version 2.1.0 software. The data also indicates that there was around 19% of reads corresponding to miRNAs sequences.

Supplementary Table 4S: Normalized miRNAs sequences: After the non-coding RNAs were identified, we showed the miRNAs normalized reads number for each sample.

Supplementary Table 5S: The miRNAs profile under each condition: Common miRNAs in all the samples (in blue) and the specific miRNAs (in red) under each condition after miRNAs normalized reads number.

Supplementary Table 6S: Significant miRNAs for each comparison of experimental groups: NGS differential expression miRNAs, the table show the fold change values and the statistics analysis value and the p-values and p-values adjusted. In red we show the miRNAs with a significant change minor of 0.05.

Supplementary Table 7S: KEGG and GO major enriched pathways: Detailed information of genes enrichment in KEGG pathways and GO terms is provided. We observe the genes related with each pathway and the statistic value.
